# p75NTR/proBDNF Modulates Basal Cell Carcinoma (BCC) Immune Microenvironment via Necroptosis Signaling Pathway

**DOI:** 10.1155/2021/6652846

**Published:** 2021-02-01

**Authors:** Qingli Lu, Yuanyuan Qu, Yuan Ding, Xiaojing Kang

**Affiliations:** Department of Dermatology and Venereology, People's Hospital of Xinjiang Uygur Autonomous Region, Urumqi 830000, China

## Abstract

Basal cell carcinoma (BCC) is the most common skin cancer. While most of the basal cell carcinomas were localized lesion and can be easily managed, the treatment options to the advanced basal cell carcinomas are still remarkably limited. In recent years, proBDNF and its receptor p75NTR have been reported to play important roles in various diseases, including cancers and psychotic disorders. However, the role of p75NTR/proBDNF signaling in basal cell carcinoma remains unclear. Here, we found that the expression level of p75NTR/proBDNF was decreased in basal cell carcinoma patient samples and cell lines. In vitro study showed overexpression of p75NTR/proBDNF could significantly facilitate tumor cell death, including inflammatory-silent apoptosis and lytic inflammatory activated necroptosis. In vivo study showed overexpression of p75NTR/proBDNF dramatically promotes tumor-associated macrophage (M1) and T cell recruitment in a syngeneic mouse model of BCC. These results show a crucial role for p75NTR/proBDNF signaling in basal cell carcinoma immune microenvironment.

## 1. Introduction

Basal cell carcinomas (BCCs) are common and costly skin cancer. The recent data point showed 5.4 million BCCs, and squamous cell carcinomas (SCCs) were diagnosed in 3.3 million Americans and showed a rising tendency [1–3]. The established BCC etiologies were environmental changes, and genetic disorders include Gorlin-Goltz syndrome and Xeroderma Pigmentosum [4]. BCCs were usually localized. Thus, the standard invasive and noninvasive procedures could achieve satisfying primary control. However, recurrence is common (10-20% recurrence rate posttreatment for five years) and might lead to a significantly worse prognosis [5]. Some subsets of BCCs, like the advanced basal cell carcinomas (aBCCs), are challenging to treat, and the prognosis is dismal. These facts made the further understanding of BCC pathogenesis became a necessary and urgent topic.

Brain-derived neurotrophic factor (BDNF) as a member of the neurotrophin family has been reported to play vital roles in nervous system development and tumorigenesis [6, 7]. Like other neurotrophins, the initially generated proBDNF could be cleaved to form mature BDNF (mBDNF) [8]. The proBDNF and mBDNF could bind to their receptor and exert opposite biological effects in the central nervous system [9, 10]. Specifically, the receptors of proBDNF are p75 pan-neurotrophin receptor (p75NTR) and sortilin, and the receptor of mBDNF is tropomyosin receptor kinase B (TrkB) [11, 12]. Except for the brain, the natural proBDNF expression has also been found in the peripheral nervous system, immune cells, and other tissues such as the skin, olfactory epithelium, and intestine [13]. In the skin, proBDNF was found in the keratinocytes and nerve fibers. In the immune cells, proBDNF and its receptor p75NTR were found to be upregulated in parasite infection and neuroinflammation [14]. The monoclonal antibody-based proBDNF blockade could significantly reduce postperipheral inflammation pain hypersensitivity [15]. The previous study also showed upregulated proBDNF in meningeal, and peripheral CD4^+^ T cells could cause immunosuppressive environment, thus, to promote the pathogenesis of sepsis-associated encephalopathy [13]. Evidence mentioned above showed the possibility that the proBDNF could interact with BCC by immune microenvironment regulation.

To explore the roles of proBDNF in BCC immune microenvironment regulation, we performed this in vivo and in vitro study. We found that proBDNF/p75NTR overexpression could modulate BCC immune microenvironment. Specifically, proBDNF/p75NTR overexpression upregulated tumor-associated macrophage recruitment and downregulated neutrophil recruitment. In addition to innate immune cells, proBDNF/p75NTR overexpression dramatically increased the level of CD8 T cells. The proBDNF/p75NTR could modulate immune cell recruitment through the necroptosis signaling pathway.

## 2. Results

### 2.1. The Expression Level of proBDNF/p75NTR in BCC Patient Samples, Cell Lines, and Adjacent Noncancerous Tissues

**Figure 1:**
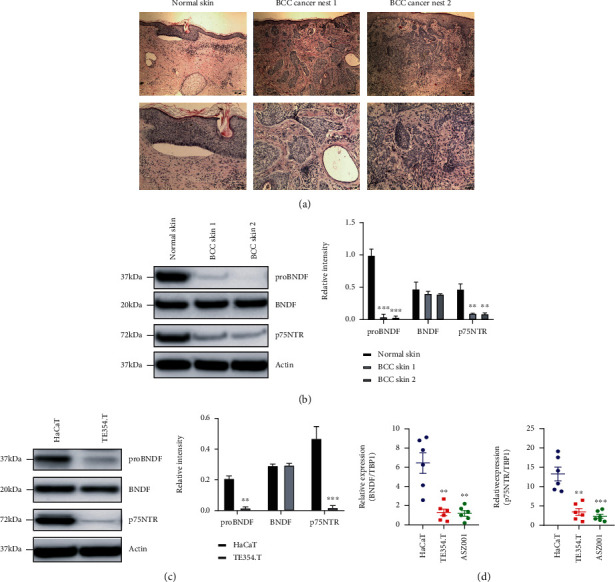
The expression level of proBDNF/p75NTR is downregulated in the BCC patients and cell lines. (a) Histology analysis in primary BCC tissues compared to normal skin samples. (b) Compared to the normal skin, the protein expression levels of p75NTR/proBDNF in BCC samples were significantly downregulated. ^∗^*P* < 0.01, ^∗∗^*P* < 0.01, ^∗∗∗^*P* < 0.001. (c, d) Compared to HaCaT cell, the protein expression levels and transcriptional levels of p75NTR/proBDNF in TE354.T and AZS001 cell lines were significantly downregulated. ^∗^*P* < 0.01, ^∗∗^*P* < 0.01, ^∗∗∗^*P* < 0.001.

To investigate the potential roles of proBDNF/p75NTR in BCC progression, the expression levels of proBDNF/p75NTR in BCC and control were evaluated by using western blotting. As shown in Figure 1(a), the classical BCC pathological structures include basaloid cells with scant cytoplasm and elongated hyperchromatic nuclei, peripheral palisading, peritumoral clefting, and mucinous alteration of surrounding stroma could be observed in BCC cancer nest. As shown in Figure 1(b), proBDNF and its receptor p75NTR were dramatically downregulated in BCC patient samples in comparison with adjacent noncancerous tissues (ANCTs). As shown in Figure 1(c), we also investigated the expression of proBDNF and its receptor p75NTR in BCC cell line TE354.T and immortalized human keratinocytes HaCaT cells. The proBDNF and its receptor p75NTR were dramatically reduced in TE354.T cell when compared with HaCaT cell. As shown in Figure 1(d), the mRNA level of BDNF and its receptor p75NTR are also dramatically reduced in TE354.T as well as ASZ001 cell lines. These findings suggested that proBDNF and its receptor p75NTR could be a potential BCC suppressor.

### 2.2. The Regulation Role of p75NTR/proBDNF Expression in BCC Cell Proliferation and Cell Death

**Figure 2:**
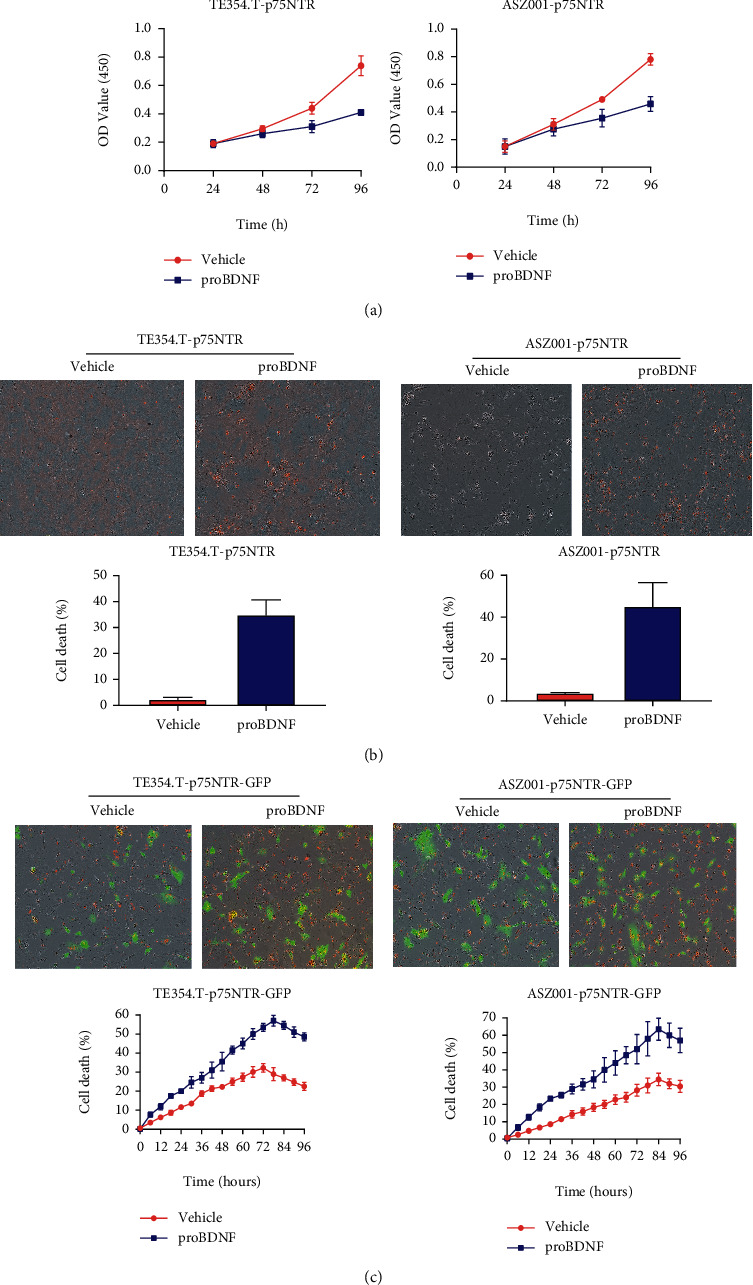
p75NTR/proBDNF regulates BCC cell proliferation and cell death. BCC cells stabilized expressed p75NTR recombinant GFP were treated with proBDNF. Cell proliferation was determined by MTT assay. (b) Cell death (apoptosis) was tracked by staining with Annexin V/PI kit. (c) Cell death (necroptosis) was tracked by staining with cytotoxic red.

Since p75NTR/proBDNF was downregulated in BCC patient samples and cell lines, we sought to explore the regulation role of p75NTR/proBDNF expression in BCC cell proliferation and cell death. We generated stable BCC cell lines TE354.T-P75NTR and ASZ001-P75NTR and treated with the proBDNF-containing medium for 96 hours. As shown in Figure 2(a), adding of proBDNF significantly inhibited the cell proliferation in both TE354.T-P75NTR and ASZ001-P75NTR cells. Since p75NTR/proBDNF has been reported to interact with the apoptosis process, we tested the cell viability of TE354.T-P75NTR and ASZ001-P75NTR cells with standard Annexin V/PI staining. As shown in Figure 2(b), more cells underwent apoptosis in p75NTR/proBDNF overexpressed cells. Apart from inflammation silent death, we also evaluated lytic inflammatory cell death like necroptosis. We generated stable BCC cell lines that express p75NTR with the fusion of a GFP cDNA controlled by an inducible promoter. The cell death was analyzed by tracking cytotoxic red signal using Incucyte live-cell imaging system. As shown in Figure 2(c), overexpression of p75NTR/proBDNF dramatically increased the TNF-induced necroptosis compared with vehicle control, which is manifested by the enhanced red signal. Abovementioned results suggested that p75NTR/proBDNF played a function in controlling BCC cell proliferation and cell death, including apoptosis and necroptosis.

### 2.3. p75NTR/proBDNF Controls RIPK1/RIPK3-Mediated Necroptosis

**Figure 3:**
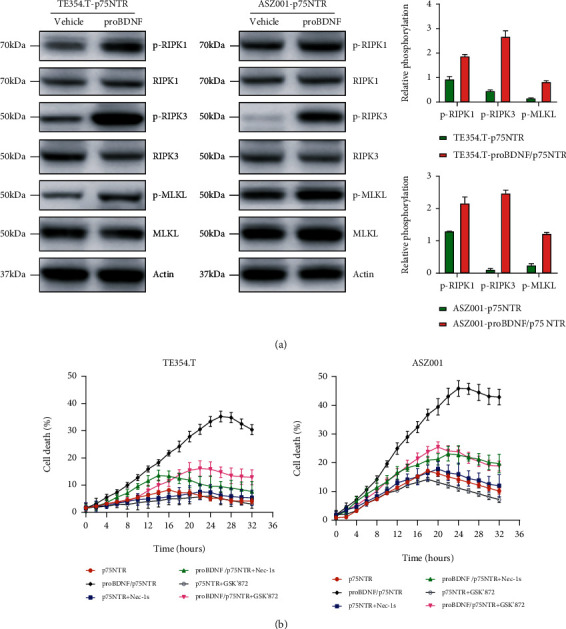
p75NTR/proBDNF controls RIPK1/RIPK3-induced necroptosis. (a) Western blot analysis of p-RIPK1, p-RIPK3, and p-MLKL protein levels inTE354.T-P75NTR and ASZ010-p75NTR cells treated with or without proBDNF plus 20 ng/mL TNF. (b) TE354.T-P75NTR and ASZ010-p75NTR cells stabilized expressed with fused GFP were treated with or without proBDNF plus 20 ng/mL TNF. Necroptotic cell death was monitored by staining with cytotoxic red and monitored by Incucyte.

Necroptosis is widely viewed as an inflammatory cell death. The role of necroptosis in cancer development is still mysterious. In order to determine whether p75NTR/proBDNF modulates TNF-induced necroptosis, we stably overexpressed p75NTR in both TE354.T and ASZ001 cell line that express a GFP reporter gene controlled by inducible promoter. The necroptosis key adaptor proteins RIPK1 and RIPK3 as well as their downstream effector protein MLKL were determined. As showed in Figure 3(a), compared with vehicle group, add of proBDNF dramatically increases the level of p-RIPK1, p-RIPK3, and p-MLKL. Correspondingly, the cell death was analyzed by tracking cytotoxic red signal using live image system Incucyte. As showed in Figure 3(b), compared with vehicle group, add of proBDNF dramatically increases TNF-induced cell death, which is manifested by enhanced red signal. To analyze whether p75NTR/proBDNF overexpression induces RIPK1/RIPK3-mediated necroptosis, we treated cells with RIPK1 inhibitor Nec-1s and RIPK3 inhibitor GSK′872. As showed in Figure 3(b), either RIPK1 or RIPK3 inhibition via Nec-1s and GSK′872 efficiently reverses p75NTR/proBDNF-induced cell death. Overall, these data suggest that p75NTR/proBDNF is critical to regulate RIPK1/RIPK3-mediated necroptosis.

### 2.4. Human and Mouse Model BCC Samples Exhibited Increased Macrophage Infiltration Compared to Normal Skin

**Figure 4:**
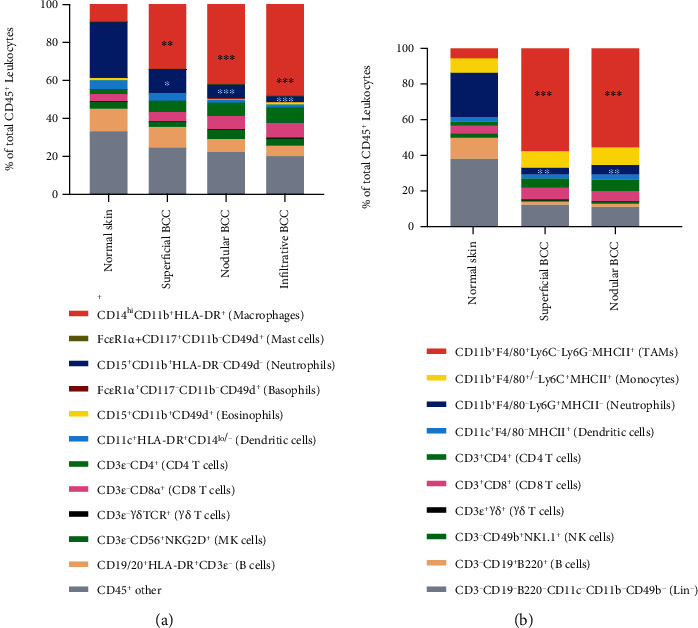
High macrophage density and lower neutrophil density in human BCC skin and a syngeneic mouse model of BCC. (a) In human samples, FACS analysis of leukocytes as a % of total CD45^+^ cells in normal skin, the various grade of BCC. ^∗∗^*P* < 0.01 compared to normal skin, ^∗∗∗^*P* < 0.001 compared to normal skin by Mann–Whitney test. (b) In a syngeneic mouse model of BCC, FACS analysis of leukocytes as a % of total CD45^+^ cells in normal skin, various grade of BCC. ^∗∗^*P* < 0.01 compared to normal skin, ^∗∗∗^*P* < 0.001 compared to normal skin by Mann–Whitney test.

To explore how the tumor immune microenvironment may contribute to the treatment resistance of BCC, we profiled the immune cell infiltration by performing polychromatic (12-color) fluorescent-activated cell sorting (FACS) on patient samples and mouse model samples. As shown in Figure 4(a), we found increased CD14^+^CD11b^+^HLA-DR^+^ monocytes/macrophages ratio in human BCC samples compared to normal skin tissue. We also observed a decreased CD15^+^CD11b^+^HLA-DR^−^CD49^−^ cells (neutrophils) ratio in different grade of human BCC samples compared to normal skin. No other immune cell ratio showed intergroup difference. In mouse models, we tested K14CreER/SmoM2 and K14CreER/Ptch1KO transgenic mice, which express a constitutively active smoothened mutant protein SmoM2 in the basal keratinocytes upon tamoxifen treatment, resulting in ligand-independent activation of Hedgehog signaling. As shown in Figure 4(b), the similar alteration of immune cell infiltration pattern could be observed. An increased CD11b^+^CD14^+^HLA-DR^+^ monocytes/macrophages ratio and a decreased CD11b^+^CD15^+^HLA-DR^−^CD49^−^ cells (neutrophils) ratio in mouse model BCC samples compared to the control group. These results indicate that compared to normal skin, BCCs are highly infiltrated by CD45^+^ immune cells, with the predominant CD45^+^ immune cell population being CD11b^+^CD14^+^CSF-1R^+^ monocyte/macrophage lineage cells.

### 2.5. The p75NTR/proBDNF Overexpression Modulates Immune Microenvironment in Mouse Models

**Figure 5:**
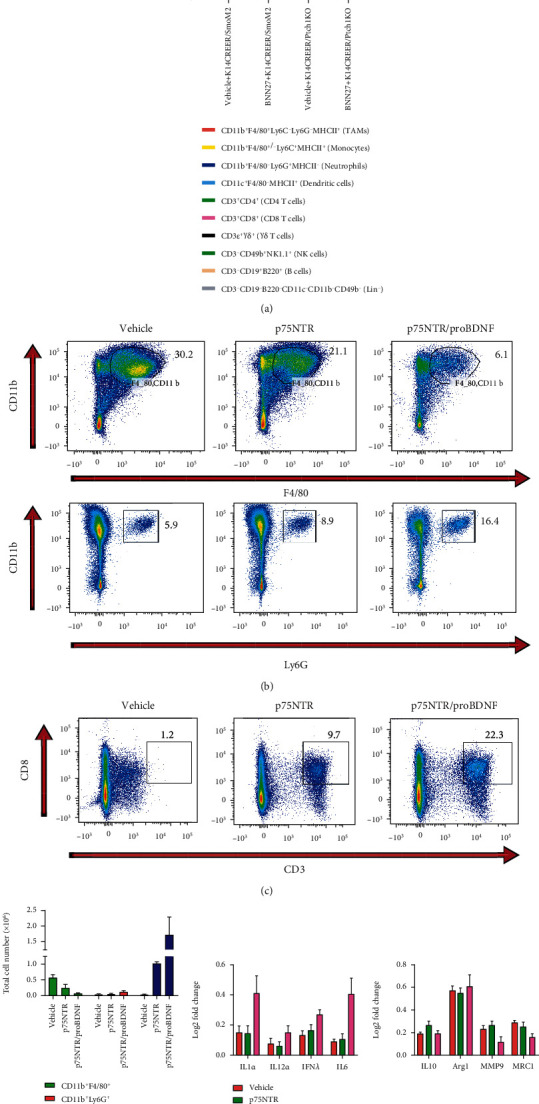
p75NTR/proBDNF overexpression modulates the recruitment of adaptive and innate immune cells. (a) Representative gating (FlowJo) and quantitation of CD45^+^CD11b^+^F4/80^+^ cells (macrophages) and CD45^+^CD11b^+^Ly6G^+^ cells (neutrophils) in normal skin, p75NTR BCC, and p75NTR/proBDNF BCC. (b–d) Representative gating (FlowJo) and quantitation of CD45^+^CD3^+^CD8^+^ cells (CD8 T cells) in normal skin, p75NTR BCC, and p75NTR/proBDNF BCC. (e) In human samples, quantitation of cytokines and chemokines of M1 and M2 (macrophages) in normal skin and p75NTR/proBDNF BCC. In a syngeneic mouse model of BCC, quantitation of cytokines and chemokines of M1and M2 (macrophages) in normal skin and p75NTR/proBDNF BCC.

It was widely accepted that p75NTR/proBDNF could play a predominant role in regulating immune microenvironment during tumor progression. To explore if the p75NTR/proBDNF's regulation role exists in BCC immune microenvironment, we investigated biopsies from p75NTR agonist BNN27 treated BCC mouse model. As shown in Figures 5(a)–5(d), the NK cells (CD3-CD49b^+^NK1.1^+^), B cells (CD3^−^CD19^+^B220^+^), and T cells (CD3^+^TCR^+^) showed no intergroup statistical difference. The overexpression of p75NTR/proBDNF could dramatically change the macrophage, neutrophils, and CD8^+^T cell infiltration. Specifically, downregulated tumor associate macrophage (CD11b^+^F4/80^+^Ly6C^−^Ly6G^−^MHCII^+^), upregulated neutrophils (CD11b^+^F4/80^+/-^Ly6C^+^MHCII^+^), and upregulated CD8 T cells (CD3^+^CD8^+^) could be observed in the p75NTR/proBDNF overexpression group.

Theoretically, activated macrophages would be polarized into two different phenotypes and perform distinct roles in the immune system. Classically activated macrophages (M1 polarization) could mediate inflammatory responses and increase the tumoricidal capacity. Alternatively, activated macrophages (M2 polarization) could contribute to tissue repair, tumor progression, and persistent infection. To explore the macrophage polarization activity in p75NTR/proBDNF overexpression condition, we analyzed the M1 and M2 specific cytokines. As shown in Figure 5(e), the M1-like instead of M2-like polarization rate alteration was detected. These results showed that p75NTR/proBDNF has a predominant role in regulating BCC immune microenvironment.

## 3. Materials and Methods

### 3.1. Ethics Statement

Our experiment protocol designed was approved by the Ethics Committee of the People's Hospital of Xinjiang Uygur Autonomous Region.

### 3.2. Human Tumor and Normal Skin Tissue Procurement

Human BCC samples were obtained from surgeries performed at the Department of Dermatology and Venereology, People's Hospital of Xinjiang Uygur Autonomous Region. Normal skin samples were harvested from freshly resected skin tissues declined for transplantation at the Department of Dermatology and Venereology, People's Hospital of Xinjiang Uygur Autonomous Region. All tissues were processed within 24 hours of resection. All human tissues were used without identifiers under an approved Committee on Human Research (CHR) protocol.

### 3.3. Preclinical Mouse Models and Animal Husbandry

All mice were maintained within the People's Hospital of Xinjiang Uygur Autonomous Region for Animal Care barrier facility according to IACUC procedures. The animal facility was managed according to IACUC procedures. To generate orthotopic BCC tumors, cell lines less than ten passages from lentiviral infection of reporter genes were grown in plastic T-150 Petri dishes to 80% confluence, at which point they were harvested by trypsin digestion, washed 1x in sterile PBS and resuspended in sterile PBS at a final concentration of 10^7^ cells/mL and placed on ice. 2 × 10^6^ cells were injected i.p. into wild-type male C57BL/6 mice (Charles River) that were 6-12 weeks of age.

### 3.4. Generation of Lentiviruses

To produce construct of p75NTR with the green fluorescent protein (GFP), the pLKO.1 vector containing the puromycin resistance gene was used. To generate lentiviruses, we coexpressed VSV-G and delta-8.9 in HEK-293T cells and then purified using PEG-it (System Biosciences).

### 3.5. Western Blotting

To test necroptosis adaptor proteins using immunoblot, we collect cell pellets by trypsin digestion and lysis them using RIPA buffer. The total protein concentration was determined using BCA protein assay kit. The samples were run by using 4%–12% polyacrylamide gels. After electrophoretic protein is transferred onto NC membranes with a Trans-Blot® Turbo™ Transfer System (Bio-Rad). We block the nonspecific binding by incubation with 5% nonfat milk and then incubate membranes with following primary antibodies, including proBDNF (57220, Cell Signaling Technology (CST)), BDNF (95702, CST), p75NTR (74921, CST), and Actin (37705, CST). Membranes were washed forth and incubated with the HRP-conjugated secondary antibodies (7076, anti-mouse IgG; 7074, anti-rabbit IgG). The interested proteins were imaged with chemiluminescence (Millipore, Billerica, MA, USA).

### 3.6. Gene Expression Analysis

Total RNA was collected using RNeasy Mini Kit (74104, QIAGEN) according to the manufacturer's instructions. The extracted RNA was transcribed reversely into cDNA using a First-Strand cDNA Synthesis Kit (4368814, Applied Biosystems). Real-time PCR was performed using an Applied Biosystems 7900HT Fast Real-Time PCR machine by using SYBR green PCR master mix (Applied Biosystems).

### 3.7. Cell Death Assay

HaCaT, TE354.T, and ASZ001 were grown and cultured as demonstrated in ATCC. To determine the cell death, cells were plated in 48-well plates and grown to 80% confluence. Cells were then stimulated with 20 ng/mL TNF (T6674, Millipore Sigma) and tracked using cell death marker—cytotoxic red (4632, Essen Bioscience Inc.). The plate was scanned every 2 hours. The images were analyzed using the IncuCyte analyzer (Essen Bioscience).

### 3.8. Flow Cytometry

For human samples, single cells from malignant BCC and normal skin tissues were prepared as follows: tissues were minced completely and then lysed at 37°C for 45 minutes in a mixed solution of 2.0 mg/mL Collagenase A (Roche) and 50 units/mL DNase I (Roche). Cells were then filtered by a 70 *μ*m nylon filter and centrifuged at 1,200 rpm × 5 minutes, followed by washing with PBS (1x). Two million cells were then incubated in a solution consisting of a 1 : 20 dilution of Fc Receptor Binding Inhibitor (eBioscience) and 1 : 500 Live/Dead Aqua stain (Invitrogen) diluted in PBS. Cells were then incubated with fluorescently labeled monoclonal antibodies as previously described in a FACS buffer (2% FBS in DPBS). After 30 min incubation at 4°C, cells were washed 1x with FACS buffer and fixed with 4% formalin for 30 min at 4°C, washed 1x with FACS buffer and then resuspended in FACS buffer. Samples were run on an LSRII flow cytometer (BD Biosciences). Gating strategy to identify specific immune cell populations was performed using the FlowJo software v9.5. Statistical analysis was performed using the GraphPad Prism software.

For mouse samples, single cells from malignant BCC and normal skin tissues were prepared as described above. Cells were incubated in PBS + rat anti-mouse CD16/CD32 mAB (BD Biosciences) 1 : 200 + Live/Dead Aqua stain (Invitrogen) at 1 : 500 on ice for 30 min. Cells were then incubated for 30 min at 4°C with fluorophore-conjugated anti-mouse monoclonal antibodies at the manufacturer's recommended concentration diluted in FACS buffer: CD45-PE-Cy7 (30-F11, eBioscience), CD3e-PerCP710 (17A2, eBioscience), CD69-FITC (H1.2F3, Biolegend), CD4-PE (GK1.5, eBioscience), CD25-APC-780 (PC61.5, eBioscience), CD8a-APC (53-6.7, eBioscience), *γδ*TCR-PE-Cy5 (GL3, eBioscience), NK1.1-PE (PK136, eBioscience), CD49b-APC (DX5, eBioscience), CD49b-PerCP-Cy5.5 (DX5, Biolegend), CD19-Alexa700 (6D5, eBioscience), CD19-PerCP-Cy5.5 (6D5, Biolegend), B220-Qdot655 (RA3-6B2, eBioscience), CD11b-Alexa700 (M1/70, eBioscience), CD11c-APC-780 (N418, ebiocience), Ly6C-APC (N418, eBioscience), Ly6G-PE (1A8, eBioscience), CD206-FITC (MR5D3, eBioscience), PDCA-1-PE (eBio927, eBioscience), and CD103-FITC (2E7, Biolegend). After 30 min incubation at 4°C, cells were washed 1x with FACS buffer and fixed with 4% formalin for 30 min at 4°C, washed 1x with FACS buffer and then resuspended in FACS buffer.

## 4. Discussion

The current clinical difficulties of BCCs are mainly about advanced BCCs, which includes metastatic BCCs and local refractory BCCs [16]. Although these cases were regarded as rare malignancy, the dismal prognosis still requires considerable clinical and basic science attention. From the clinical aspect, multidisciplinary tumor board consultation is strongly suggested by various publications in recent years. Local treatment options and systemic treatment options include platinum-based chemotherapy, and smoothened inhibitors should be selected accordingly [17, 18]. However, objective treatment criteria or strong evidence-based medicine recommendations were lacked until now. The popular smoothened inhibitor, Vismodegib, was also limited by its obvious adverse effects [19, 20]. These facts made the further biological understanding of aBCC is still greatly needed. In this study, we found downregulated proBDNF/p75NTR expression in BCC samples and cell lines. In vitro study showed adding of external proBDNF could inhibit BCC tumorigenesis. Moreover, proBDNF/p75NTR overexpression could lead to increased BCC apoptosis and necroptosis activity. Since the previous study showed, proBDNF/p75NTR signaling is an important immune-related regulator. We profiled BCC immune cell infiltration and found that macrophage is one of the BCC-specific infiltrated immune cells. The proBDNF/p75NTR overexpression was correlated with increased M1-like polarization rate but nonalternated M2-like polarization.

BDNF, as an important member of the neurotrophic factor family, is a protein synthesized in the brain and widely distributed in the central and peripheral nervous system [6, 21]. BDNF has been extensively involved in multiple physiological processes including brain development, homeostasis, and immune regulation [10, 22]. Interruption of physiological BDNF function was correlated with neurodegeneration, neuropathic pain, psychiatric disorder, breast cancer, ovarian cancer, and gastric cancer [23–26]. However, the role of BDNF in skin cancer remains unknown. Our results showed that proBDNF/p75NTR could be a BCC suppressor. Furthermore, external proBDNF treatment could reverse BCC tumorigenesis.

Necroptosis is a kind of lytic inflammatory cell death. In cancer, necroptosis was reported to function as an antitumoral activity [27, 28]. Necroptosis response could be found in cancer initiation, progression, and metastasis [27–29]. However, necroptosis also suppresses the myeloid cell-mediated adaptive immune response and thus facilitates cancer progress [30, 31]. One recent study indicated that enhance the immunogenicity of dying cells within the tumor microenvironment through specific activation of the necroptotic pathway represents an efficient therapeutic strategy that could warrant further clinical application [31].

Our study demonstrated that p75NTR/proBDNF is critical to control immune microenvironment during BCC progression. This immune-related function of p75NTR/proBDNF is closely linked with their role in regulating necroptosis.

## Data Availability

The data used to support the findings of this study are available from the corresponding author upon request.
